# Health-related quality of life disparities among Hispanic/Latinx patients with nephrolithiasis

**DOI:** 10.1007/s00240-023-01414-w

**Published:** 2023-03-15

**Authors:** Alec R. Flores, Garen Abedi, Carol B. Girgiss, Jonathan H. Berger, Kristina L. Penniston, Shuang Li, David F. Friedlander, Seth K. Bechis, Roger L. Sur

**Affiliations:** 1 Department of Urology, UC San Diego Health, San Diego, USA; 2 Department of Urology, University of Wisconsin, Madison, USA; 3 Department of Urology, University of North Carolina, Chapel Hill, USA

**Keywords:** Wisconsin quality of life questionnaire, Quality of life, Nephrolithiasis, Urolithiasis, Health disparities, Latinx, Hispanic

## Abstract

It is documented that Hispanic/Latinx kidney stone formers have inferior health-related quality of life (HRQoL) compared to the general population. We hypothesized that socioeconomic factors drive HRQoL disparities. Specifically, we sought to determine if medical insurance type is associated with HRQoL disparities among Hispanic/Latinx stone formers. This was a prospective cohort observational study of patients with kidney stones across the University of San Diego Health Care System. Patients enrolled from June 2018 to August 2020 completed a validated Wisconsin Stone Quality of Life questionnaire (WISQoL). Patient characteristics and self-reported HRQoL were compared between Hispanic/Latinx and non-Hispanic/Latinx stone formers using MANCOVA and ordinal logistic regression. Matched group comparisons were performed based on age, gender, body mass index, stone symptoms, and insurance type using MACOVA. A total of 270 patients were enrolled (Hispanic/Latinx *n* = 88; non-Hispanic/Latinx *n* = 182). Hispanic/Latinx stone formers had higher rates of public insurance at baseline (*p* < 0.001) with significantly lower HRQoL [social impact (*p* = 0.007)]. However, a matched cohort comparison demonstrated no differences. On multivariate analysis, private insurance increased the likelihood of having higher HRQoL (OR 2.21, *p* = 0.021), while stone symptoms (OR = 0.06, *p* < 0.001) and emergency department visits (OR = 0.04, *p* = 0.008) decreased chances of higher HRQoL. Ethnicity was not a significant factor in HRQoL scores on multivariate analysis. Our analysis suggests that differences in HRQoL among Hispanic/Latinx stone formers may be primarily driven by socioeconomic factors as opposed to clinical or racial differences. Specifically, source of insurance appears to have significant effect on HRQoL in this ethnic group.

## Introduction

In the United States, healthcare disparities are widespread for Hispanic, Latinx, and other ethnic minority groups [1, 2]. Currently representing the second largest demographic group in prevalence and incidence rates of nephrolithiasis, Hispanic/Latinx kidney stone formers have been well documented in prior studies to have inferior quality of life compared to the general population [3–5]. However, to our knowledge, no prior study has sought to examine the underlying variables that may explain these disparities within the kidney stone patient population.

One integrative component of complex socioeconomic health determinants is health insurance, which has been identified as a possible predictor of health care disparities [6]. Importantly, reliance on public health insurance or absence of any form of insurance is more common among racial minorities [7]. The quality or lack of health insurance affects access to care and basic preventive health interventions. For example, lower quality health insurance limits choices in providers, obstructs continuity of care (including access to specialty providers), and can contribute to inconsistent medication adherence. In fact, despite being the largest minority group in the United States, Hispanic/Latinx patients are disproportionately more likely to be underinsured or completely uninsured [8]. These circumstances can ultimately help explain higher incidence of untreated chronic medical conditions, inferior health outcomes and quality of life in this population.

As a result, we hypothesized that socioeconomic factors are primary drivers of these differences in HRQoL. Specifically, we sought to determine if medical insurance type was a predictor of HRQoL disparities among Hispanic/Latinx stone formers.

## Methods

### Study design

This was a prospective cohort observational study of patients with kidney stones at UC San Diego Health Care System who were enrolled over a 2-year period from June 2018 to August 2020. The protocol was approved by the institutional review board (IRB # 140519) and all patients provided informed written consent. Patients eligible for enrollment were legal adults (ex. 18 years of age or older), had a history of kidney stones (ex. current or previous stone), had no history of neurological or neurodevelopmental impairment, and spoke English or Spanish. Patients could either be planned surgical candidates, prior surgical patients, or patients followed conservatively. All patients were offered enrollment if they met inclusion criteria.

### Quality of life measure

Participants completed a validated English or UC San Diego approved Spanish of the Wisconsin quality of life (WISQoL) questionnaire at their enrollment visit based on their language preference. Pre-operative WISQoL questionnaire data were collected from subjects. The WISQoL is a validated 28-item tool to assess stone disease-specific quality of life in the last month across 4 domains, including social, symptom impact, emotion, and vitality. Items are rated on a 5-point Likert scale from very true (1 point) to not at all true (5 points) with a score of 5 corresponding to higher quality of life [9]. An overall quality of life score was calculated as the average rating. Subdomain scores were calculated similarly.

### Clinical and sociodemographic characteristics

Following written consent, patient records were reviewed to obtain additional data, including age, sex, body mass index (BMI), medical comorbidities, stone burden, insurance information, household ZIP code, and details regarding the medical management of stone disease (specifically diuretics or alkalinizing therapy). Race and ethnicity were defined by self-report.

Variables including BMI were converted to categorical variables using clinically meaningful cutoffs. BMI was defined using adult BMI categories from the Centers for Disease Control and Prevention with an additional super-obese category for BMI greater than 40 kg/m^2^. Insurance information was defined using private plan (ex. employment-based, direct-purchase, TRICARE) versus public plan (ex. Medicare, Medicaid, VA, CHAMPVA) from the United States Census Bureau. Of note, current TRICARE has evolved into pseudo-prioritized, private like insurance (providers are community based vice military providers) for dependents, as access to military treatment facilities no longer exists.

### Statistical analysis

We observed that standardized scores were not normally distributed; therefore, cutoffs of 20, 40, 60, and 80 on the standardized scale were used to form the ordered categories. Ordinal logistic regression analysis was performed to explore the association between demographics, clinical factors, and quality of life (QOL) scores.

Variables examined included age, sex, BMI, ethnicity, presence of comorbidities, distance to care (miles), and total stone burden (longest mm in either axial or coronal views). Comorbidities recorded included gastro-esophageal reflux, hyperlipidemia, coronary vascular disease, diabetes, hypertension, lower urinary tract symptoms, urinary tract infections, osteoporosis, depression, anxiety, and musculoskeletal disease. Charlson Comorbidity Index was then calculated based on these data. Additional variables included self-reported stone-related symptoms, emergency department visits, hospitalizations, education, income, and insurance type (private vs. public). Using MANCOVA 2.1, mean domain scores of WISQoL questionnaire were compared between White non-Hispanic/Latinx and White Hispanic/Latinx. Analysis was repeated after match pair comparison was performed while controlling for significant variables including age, sex, BMI, self-reported stone-related symptoms, emergency department visits, and insurance type using MANOVA.

Demographic and clinical factors were compared using Chi-square and T test analyses. Statistics were performed using SPSS version 25. *p* < 0.05 was considered statistically significant.

## Results

270 patients were evaluated during the course of this study including 135 males and 135 females. Baseline characteristics are shown in Table 1. Patients who were identified as Hispanic/Latinx were younger, more frequently public insured (*p* < 0.001), and had higher rates of stone symptoms and obesity (*p* < 0.001 and *p* = 0.003, respectively). There was no difference in stone burden between the groups. 86.4% of patients in the Hispanic/Latinx group filled out the questionnaire at a follow-up visit versus 94.0% of patients in the non-Hispanic/Latinx group. The remainder of patients filled out the questionnaire in a preoperative setting (13.6% of Latinx/Hispanic group vs. 6.0% non-Hispanic/Latinx group) *p* = 0.036). Of those patients who filled out the questionnaires at follow-up visit, 44.7% of patients in the Hispanic/Latinx group filled out the questionnaire in a post-operative setting versus 62.6% in the non-Hispanic/Latinx group (*p* = 0.009).

**Table 1:** Baseline characteristics

	Non-Hispanic/Latinx (*N* = 182)	Hispanic/Latinx (*N* = 88)	*p*^ξ^
Age	57.9 ± 16.1	46.5 ± 14.7	** < 0.001**
Gender (men)	104 (51.1%)	33 (39.0%)	**0.002**
Insurance type (private)	127 (58.4%)	28 (32.5%)	** < 0.001**
Symptomatic (yes)	66 (40.3%)	56 (64.9%)	** < 0.001**
Obese (BMI > = 30)	57 (34.4%)	44 (49.4%)	**0.003**
BMI (kg/m^2^)	28.4 ± 7.4	31.8 ± 8.5	**0.001**
Mean Charlson comorbidity score (range)	0.39 (0–3)	0.43 (0–3)	0.257
Medical stone prevention therapy	44 (24.2%)	7 (8.0%)	**0.001**
Total stone burden (mm) on CT	12.8 ± 17.8	15.8 ± 24.2	0.272
Pre-operative candidate	12 (6%)	12 (13%)	0.05
Post-operative candidate	61 (32%)	25 (28%)	0.398
Conservative candidate	109 (58%)	51 (58%)	0.761

^ξ^*p* values are from Chi-square and *t*-tests

On MANCOVA testing, Hispanic/Latinx patients had significantly lower social impact scores in a non-matched pair comparison (76.2 vs. 59.1 *p* = 0.007) (Table 2). Hispanic/Latinx patients also had lower emotional impact scores, but were not statistically significant (66 vs 47.6, *p* = 0.05). On ordinal logistic regression analysis, patients with private insurance had significantly higher quality of life scores (OR 2.2, 95% CI 1.11–4.02 *p* = 0.021). This was similar for all domains (Table 3). There were no significant differences in HRQoL among patients from various education and income levels. Presence of stone-related symptoms and emergency room visits significantly decreased risk of higher quality of life score (OR 0.06, 95% CI 0.03–0.12 and OR 0.42, 95% CI 0.22–0.80, *p* < 0.001 and *p* = 0.01, respectively). Matched case analysis found no significant difference (Table 4).

**Table 2:** Unmatched comparison of WISQoL scores between White and Latinx populations

	Non-Hispanic/Latinx (*n* = 182)	Hispanic/Latinx (*n* = 88)	*p*^ξ^
Total score	70.2 ± 26.9	55.6 ± 29.6	0.055
Social impact	76.2 ± 30.5	59.1 ± 33.7	**0.007**
Emotional impact	66 ± 31.6	47.6 ± 33.7	0.050
Disease impact	67 ± 29.6	55.4 ± 32.2	0.416
Impact on vitality	64.7 ± 34.9	49.6 ± 36.9	0.184

^ξ^*p* values are from MANCOVA

**Table 3:** Multivariate analysis of factors effecting WISQoL scores

	Total score		Social impact		Emotional impact		Disease impact		Impact on vitality	
	OR (95% CI)	*p*	OR (95% CI)	*p*	OR (95% CI)	*p*	OR (95% CI)	*p*	OR (95% CI)	*p*
Age	0.99 (0.97–1.02)	0.74	0.98 (0.96–1.01)	0.31	1.00 (0.98–1.03)	0.51	1.00 (0.98–1.02)	0.74	1.00 (0.97–1.02)	0.98
BMI	0.97 (0.94–1.01)	0.16	0.98 (0.94–1.01)	0.32	0.98 (0.94–1.01)	0.27	0.97 (0.94–1.01)	0.20	0.98 (0.95–1.02)	0.44
Distance to care	1.00 (0.99–1.00)	0.60	1.00 (0.99–1.00)	0.38	1.00 (0.99–1.00)	0.78	1.00 (0.99–1.00)	0.60	1.00 (1.00–1.00)	0.10
Comorbidities Ref: none	0.89 (0.72–1.09)	0.27	0.91 (0.73–1.13)	0.40	0.92 (0.75–1.13)	0.45	0.87 (0.71–1.07)	0.20	0.75 (0.61–0.92)	**0.01**
Total stone burden	0.99 (0.98–1.00)	0.45	0.99 (0.97–1.00)	0.31	0.99 (0.98–1.01)	0.59	0.98 (0.97–1.00)	0.15	1.00 (0.99–1.01)	0.53
Gender Ref: female	1.55 (0.84–2.86)	0.15	1.71 (0.89–3.27)	0.10	1.31 (0.73–2.35)	0.35	0.94 (0.52–1.68)	0.83	2.09 (1.17–3.74)	**0.01**
Ethnicity Ref: white	0.97 (0.49–1.91)	0.92	0.84 (0.41–1.71)	0.63	0.82 (0.42–1.59)	0.56	0.99 (0.51–1.92)	0.98	0.81 (0.42–1.56)	0.53
Insurance Ref: Public	2.21 (1.11–4.02)	**0.02**	2.03 (1.03–3.99)	**0.03**	1.76 (0.95–3.27)	0.07	1.86 (1.00–3.44)	**0.04**	1.98 (1.07–3.65)	**0.03**
Stone										
Have stone Ref: none	0.66 (0.29–1.50)	0.32	0.68 (0.27–1.69)	0.41	0.56 (0.26–1.22)	0.14	1.08 (0.51–2.29)	0.83	0.55 (0.26–1.17)	0.12
Symptoms Ref: no	0.06 (0.03–0.14)	** < 0.001**	0.09 (0.04–0.22)	** < 0.001**	0.08 (0.04–0.17)	** < 0.001**	0.09 (0.04–0.20)	** < 0.001**	0.14 (0.07–0.29)	** < 0.001**
ER Ref: none	0.42 (0.22–0.80)	**0.01**	0.28 (0.14–0.56)	** < 0.001**	0.48 (0.26–0.89)	**0.02**	0.45 (0.24–0.84)	**0.01**	0.52 (0.28–0.97)	**0.04**
Traumatic event Ref: no	0.95 (0.36–2.49)	0.92	1.23 (0.44–3.45)	0.69	0.50 (0.20–1.26)	0.14	1.07 (0.41–2.75)	0.88	0.65 (0.26–1.59)	0.35
Hospitalization Ref: no	0.79 (0.33–1.84)	0.58	0.92 (0.37–2.30)	0.87	0.95 (0.41–2.20)	0.91	0.82 (0.36–1.86)	0.63	0.79 (0.35–1.79)	0.58

*p* values are from ordinal logistic regression

**Table 4:** Matched pair comparison of quality of life scores based on age, gender, BMI, symptomatic status and insurance type

	Non-Hispanic/Latinx (*n* = 88)	Hispanic/Latinx (*n* = 88)	*p*^ξ^
Total score	60.8 ± 27.8	55.8 ± 30.1	0.332
Social impact	67.0 ± 32.9	59.1 ± 34.1	0.713
Emotional impact	55.4 ± 33.3	48.2 ± 34.4	0.223
Disease impact	57.6 ± 30.8	55.3 ± 32.7	0.797
Impact on vitality	52.9 ± 35.6	49.4 ± 37.2	0.645

^ξ^*p* values are from MANCOVA

## Discussion

In our study cohort, we found that differences in HRQoL scores were significantly associated with insurance type. Patients holding a private insurance had double the odds of having better health-related quality of life scores on WISQoL across all four domains including social impact, emotional impact, disease impact, and impact on vitality. In addition, having self-reported stone-related symptoms and emergency department visits appeared to significantly lower quality of life scores, with the latter having a statistically and clinically significant decrease in quality of life odds by 50%. Interestingly, there were no significant differences in HRQoL among patients from various education and income levels.

According to the United States Census Bureau’s 2018 Health Insurance Coverage Report, demographically 75% Caucasians, 73% Asians, 55% Blacks, and 49% Hispanic/Latinx are enrolled in private health insurance while the remainder are enrolled in public health insurance or completely uninsured [8]. Our results were similar as only 32% of Hispanic/Latinx patients had private health insurance. With respect to WISQoL, Hispanic/Latinx patients had lower social and emotional impact domain scores. However, this difference dissipated when matching the groups with respect to age, gender, BMI, self-reported stone-related symptoms, emergency department visits, and insurance type. This suggests that there may be modifiable factors at play which if addressed can potentially improve stone-related health outcomes among Hispanic/Latinx population. Racial disparities in minority groups have been investigated with regard to analgesic usage in kidney stone patients presenting to the emergency room. Berger et al. reported that Blacks and Hispanics presenting to the emergency room received fewer opioid medications and had lower incidence of ketorolac administration [10]. Given our findings and reports of others, understanding effect of insurance quality on healthcare delivery seems important in nephrolithiasis—particularly when trying to untangle effect of race.

In a prior study evaluating 2057 stone patients, Ahmad et al. reported lower quality of life scores in non-white, uninsured, low-income patients [3]. One interpretation of this study is that race confers clinical differences and hence may explain the lower quality of life. Along these lines, we wanted to know if being Hispanic/Latinx was an independent risk factor for lower quality of life. However, the above study included all minority groups and was not designed to specifically evaluate Hispanic/Latinx population.

In other disciplines, lower health-related quality of life (HRQoL) appears to be associated with public insurance [11]. In prostate cancer literature, insurance status was also associated with changes in health-related quality of life [12]. When controlling for insurance status in our match paired analysis of WISQoL scores, differences disappeared between Hispanic/Latinx and non-Hispanic/Latinx individuals. Therefore, our analysis suggests disparities are socioeconomic related, specifically insurance coverage—not inherent racial factors. Public insurance may potentially prolong the wait time for a patient to see a urologist, as specialty visits often necessitate primary care referrals. Specialty care delays logically permit exacerbation in stone burden and concomitant stone morbidity. It may also be that public insurance is a surrogate for other non-clinical factors such as patient compliance, medical insight, and perhaps language barriers.

Our study does have limitations: (1) small cohort of patients compared to prior literature, (2) questionnaires were given out at different time points in patients’ stone management, which may have introduced some recall bias especially in patients who were far removed from their painful stone episodes, (3) our dataset does not breakdown type of procedural interventions for stones which has the potential for a confounder if not equally distributed between the cohorts, (4) patients who chose to complete the surveys may have represented a higher or lower quality of life group since some patients that did not fill out the surveys.

Notwithstanding the limitations, our study sheds light on some factors that may influence quality of life in the Hispanic/Latinx kidney stone population. It is plausible that certain aspects of socioeconomic status of the patient, specifically insurance type, have a profound effect on the quality of life among Hispanic/Latinx stone formers. Given the prevalence of public insurance among this population, understanding exactly why insurance is associated with lower HRQoL remains the next logical step to advancing medical care among Hispanic/Latinx stone formers.

## Conclusions

Our study demonstrates that documented health-related quality of life disparities in the Hispanic/Latinx stone population are likely related to insurance type. This report contributes to our understanding of racial/ethnic differences in health care outcomes and creates impetus for more research into what shortcomings public insurance confers to Hispanic/Latinx kidney stone patients.

## Data Availability

The datasets analyzed during the current study are not publicly available due to institutional IRB restrictions but are available from the corresponding author on reasonable request.

## Code availability

IBM SPSS version 25 was used for statistical analysis.
